# Application of MEMS Accelerometers and Gyroscopes in Fast Steering Mirror Control Systems

**DOI:** 10.3390/s16040440

**Published:** 2016-03-25

**Authors:** Jing Tian, Wenshu Yang, Zhenming Peng, Tao Tang, Zhijun Li

**Affiliations:** 1 Institute of Optics and Electronics, Chinese Academy of Science, Chengdu 610209, China; yws@ioe.ac.cn (W.Y.); prettang@gmail.com (T.T.); zhijunhome@sina.com (Z.L.); 2 School of Opto-Electronic Information, University of Electronic Science and Technology of China, Chengdu 610054, China; zmpeng@uestc.edu.cn; 3 Key Laboratory of Optical Engineering, Chinese Academy of Sciences, Chengdu 610209, China; 4 University of Chinese Academy of Sciences, Beijing 100039, China

**Keywords:** MEMS accelerometer, MEMS gyroscope, acceleration feedback control, multi-loop control, light of sight stabilization

## Abstract

In a charge-coupled device (CCD)-based fast steering mirror (FSM) tracking control system, high control bandwidth is the most effective way to enhance the closed-loop performance. However, the control system usually suffers a great deal from mechanical resonances and time delays induced by the low sampling rate of CCDs. To meet the requirements of high precision and load restriction, fiber-optic gyroscopes (FOGs) are usually used in traditional FSM tracking control systems. In recent years, the MEMS accelerometer and gyroscope are becoming smaller and lighter and their performance have improved gradually, so that they can be used in a fast steering mirror (FSM) to realize the stabilization of the line-of-sight (LOS) of the control system. Therefore, a tentative approach to implement a CCD-based FSM tracking control system, which uses MEMS accelerometers and gyroscopes as feedback components and contains an acceleration loop, a velocity loop and a position loop, is proposed. The disturbance suppression of the proposed method is the product of the error attenuation of the acceleration loop, the velocity loop and the position loop. Extensive experimental results show that the MEMS accelerometers and gyroscopes can act the similar role as the FOG with lower cost for stabilizing the LOS of the FSM tracking control system.

## 1. Introduction

The fast steering mirror (FSM) control system is extensively applied in optoelectronic tracking equipment, which is increasingly mounted on airplanes, vessels, vehicles and other moving platforms. The traditional FSM control system usually uses fiber-optic gyroscopes (FOGs) and charge-coupled devices (CCDs) to implement strap-down stabilization. The disturbance suppression of the FSM control system is limited for its only two closed-loops [1,2,3].

In recent years, the noise of MEMS gyroscopes has been reduced with the development of research and production in MEMS industry. The noise density of the SCR1100-D02 gyroscopes used in this paper reaches 0.0085 (°/s)$/\sqrt{\text{Hz}}$ [4]. MEMS gyroscopes can be mounted on the frame of FSMs and used for the stabilization of the FSM control system as they have relatively smaller size, lower weight and lower power consumption. However, the bandwidth of MEMS gyroscopes with low noise is general less than 100 Hz, which limits the bandwidth of the velocity closed-loop and the disturbance suppression ability of the FSM control system. In order to further improve the disturbance suppression ability of the FSM control system, an acceleration loop can be implemented, so that the control system contains an acceleration closed-loop, a velocity closed-loop and a position closed-loop. Considering the installation position of the sensors is limited to the narrow spces of the reverse side of the mirror, linear MEMS accelerometers with small size, low weight and low power consumption are used in the acceleration closed-loop. A detailed description of the method about using two linear accelerometers for measuring the angular acceleration of the FSM is mentioned in [5]. The linear MEMS accelerometers used in this paper are Model 1221 units with a bandwidth above 400 Hz and noise density of 14 $\text{ug}/\sqrt{\text{Hz}}$ [6].

The acceleration feedback control (AFC) is a kind of high-precision robust control. It was proposed by Studenny and Belanger in 1984 [7] and its application in mechanical arm control was reported in a paper in 1991 [8]. In 1994, Bram de Jager studied the application of AFC in tracking control [9]. Application research on acceleration feedback performed in torque control and direct-driven mechanical arm shows that AFC is a highly effective technique [10,11]. In the previous research, the actuator was a rotary motor. In theory, the acceleration open-loop transfer function of this system driven by the rotary motor characterizes a low-pass filter. However, the actuators used in the FSM control system are voice coil motors, and the acceleration open loop transfer function includes a quadratic differential. In recent years, some scholars have used accelerometers and the focal plane array (FPA) to implement closed-loop control of the FSM [5,12]. Accelerometers have poor low-frequency response ability, while there exists a large drift in the double integral data of accelerometers, which should be corrected with the FPA, thus it is difficult to use only accelerometers in a FSM control system. The position information obtained from double integration of the accelerometers fused with FPA data was used to achieve position loop control [5]. Strictly speaking, it is a single loop position control method. Tang combined a CCD and accelerometers to implement dual closed-loop control with acceleration and position [12]. In order to avoid the saturation of double integration, the acceleration controller was designed as a bandpass filter. Therefore, there is still a quadratic differential effect within the low-frequency range, and the disturbance suppression of the FSM control system for low-frequency vibration is insufficient.

In this paper, under the conditiona of a low velocity closed-loop bandwidth, a three-closed-loop control model (MEMS accelerometer feedback, MEMS gyroscope feedback and CCD feedback) is proposed to replace the two-closed-loop control model (FOG feedback and CCD feedback) in the FSM control system. A lag controller is used to accomplish the acceleration closed-loop control. The velocity controller can be designed as a PI type controller, because the velocity open loop response with AFC exhibits mainly an integrator. This paper is organized as follows: Section 2 presents a detailed introduction to the control model of the FSM, mainly describing the CCD-based control structure and the implementation of AFC. Section 3 discusses and analyzes the performance of MEMS accelerometers and gyroscopes. Section 4 sets up the experiments used to verify this method. Concluding remarks are presented in Section 5.

## 2. Control System Model

**Figure 1:**
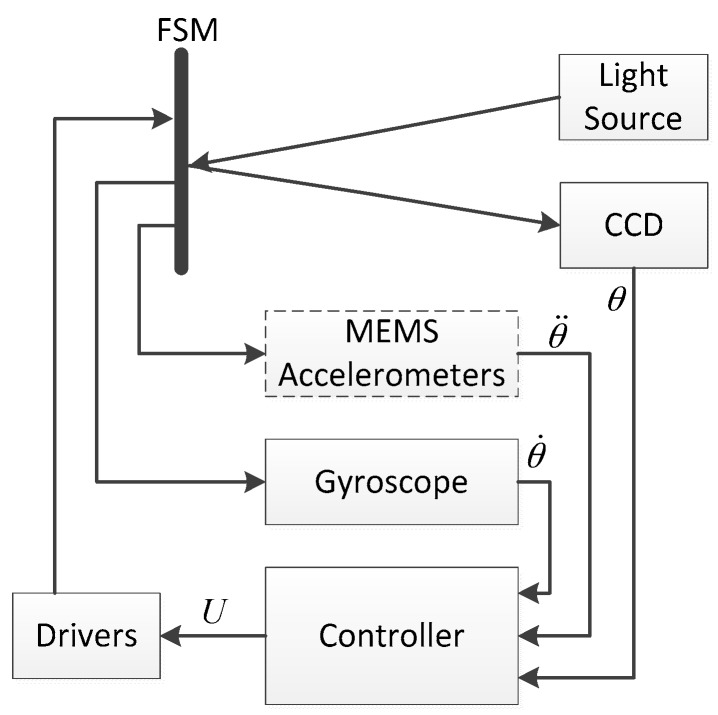
Configuration of the FSM control system.

The configuration of the FSM control system is illustrated in Figure 1. The sensors include MEMS accelerometers, fiber-optic or MEMS gyroscopes and a CCD. The controller is used to implement the control algorithm. The driver actuates the voice coil motors to achieve the stabilization control of FSM. The light source is used to simulate the target for the CCD.

The mechanical part of the FSM is a typical resonance element, while its electrical part including the drivers and the voice coil motors of the FSM is a typical first-order inertial element, which is depicted as $\frac{1}{T_{e}s+1}$. Therefore, the FSM position open loop response can be expressed as follows [12]:

(1) $$G_{p}(s)=\frac{\theta (s)}{U(s)}=\frac{K}{\frac{s^{2}}{{\varpi_{n}}^{2}}+\frac{2\xi }{\varpi_{n}}s+1}\cdot \frac{1}{T_{e}s+1}$$

The open loop natural frequency of FSMs, $\varpi_{n}$ is approximately between several Hz to tens of Hz, and the damping factor ξ is much smaller than 1 [13].

**Figure 2:**
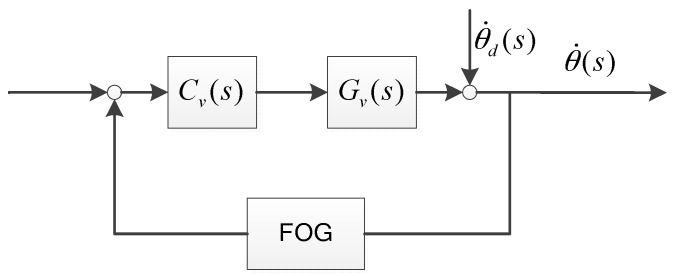
FOG control system.

The traditional FSM control system with the FOG is shown in Figure 2.

The FSM acceleration open loop response *G_v_(s)* is a differential of *G_p_(s)*, which is depicted in Equation (1):

(2) $$G_{v}(s)=\frac{\dot{\theta }(s)}{U(s)}=\frac{K}{\frac{s^{2}}{{\varpi_{n}}^{2}}+\frac{2\xi }{\varpi_{n}}s+1}\cdot \frac{s}{T_{e}s+1}$$

The velocity controller can be presented as follows:

(3) $$C_{v}\left(s\right)=G_{v}^{-1}\left(s\right)T_{low}\left(s\right)$$

*T_low_(s)* is a low-pass filter to reduce vibrations and noise. The disturbance attenuation can be depicted as follows:

(4) $$\frac{\dot{\theta }\left(s\right)}{{\dot{\theta }}_{d}\left(s\right)}=\frac{1}{1+G_{v}\left(s\right)C_{v}\left(s\right)G_{FOG}\left(s\right)}$$

**Figure 3:**
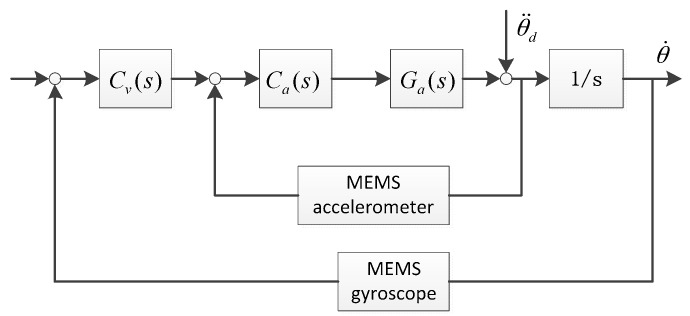
MEMS control system.

The second-order filter G_FOG_(s) represents the characteristics of the FOG [14]. The FSM control system with the MEMS accelerometers and gyroscopes is shown in Figure 3.

The FSM acceleration open loop response *G_a_(s)* is a quadratic differential of *G_p_(s)*, which is depicted in Equation (1):

(5) $$G_{a}(s)=\frac{\ddot{\theta }(s)}{U(s)}=\frac{K}{\frac{s^{2}}{\varpi_{n}^{2}}+\frac{2\xi }{\varpi_{n}}s+1}\cdot \frac{s^{2}}{T_{e}s+1}$$

The ideal acceleration controller can be designed as the inverse transfer function *G_a_(s)*. In order to increase the system gain, an integrator should be added. The ideal acceleration controller can be presented as follows:

(6) $$C_{a}(s)=K_{a}\cdot \frac{1}{s}\cdot \frac{\frac{s^{2}}{{\varpi_{n}}^{2}}+\frac{2\xi }{\varpi_{n}}s+1}{K}\cdot \frac{T_{e}s+1}{s^{2}}$$

Since the controller has complete pole-zero cancellation, the acceleration closed-loop transfer function may have high bandwidth theoretically. However, there are some disadvantages for the controller, such as the saturated double integration, the worse disturbance suppression and stability of the system induced by the inaccurate control function which is derived from inaccurate fitting of *G_a_(s)* due to the noise and measuring error of accelerometers. To avoid these deficiencies, the acceleration controller *C’_a_* is designed in Equation (7):

(7) $$C_{a}^{\prime }=\frac{K_{a}}{s}\cdot \frac{T_{e}s+1}{T_{1}s+1}$$

where *T_e_s*+1 is used to compensate phase lag. The integrator is used to compensate the quadratic differential partly. The lag element with small time constant is used to filter the high-frequency noise. The designed value of *T_1_* should be smaller than 0.01, otherwise the bandwidth of the control system will be too low. The closed-loop acceleration transfer function is expressed as follows:

(8) $$\begin{array}{cc} G_{a\_closed} & =\frac{\frac{K_{a}Ks}{\left(T_{1}s+1\right)\left(\frac{s^{2}}{{\varpi_{n}}^{2}}+\frac{2\xi }{\varpi_{n}}s+1\right)}}{1+\frac{K_{a}Ks}{\left(T_{1}s+1\right)\left(\frac{s^{2}}{{\varpi_{n}}^{2}}+\frac{2\xi }{\varpi_{n}}s+1\right)}}=\frac{K_{a}Ks}{\left(T_{1}s+1\right)\left(\frac{s^{2}}{{\varpi_{n}}^{2}}+\frac{2\xi }{\varpi_{n}}s+1\right)+K_{a}Ks}\\ & =\frac{K_{a}Ks}{\frac{T_{1}}{{\varpi_{n}}^{2}}s^{3}+\left(\frac{1}{{\varpi_{n}}^{2}}+\frac{2\xi T_{1}}{\varpi_{n}}\right)s^{2}+\left(K_{a}K+T_{1}+\frac{2\xi }{\varpi_{n}}\right)s+1} \end{array}$$

This equation is similar to a band pass filter. *G_a_closed_* is approaching zero at high frequency. On the basis of analyzing of Equations (1) and (8), the denominator of the Equation (8) can be simplified to KaKs + 1 at low frequency. And then *G_a_closed_* can be simplified as follows:

(9) $$G_{a\_closed}^{\prime }=\frac{K_{a}Ks}{\frac{T_{1}}{{\varpi_{n}}^{2}}s^{3}+\left(\frac{1}{{\varpi_{n}}^{2}}+\frac{2\xi T_{1}}{\varpi_{n}}\right)s^{2}+\left(K_{a}K+T_{1}+\frac{2\xi }{\varpi_{n}}\right)s+1}\approx \frac{K_{a}Ks}{\left(K_{a}K+T_{1}+\frac{2\xi }{\varpi_{n}}\right)s+1}\approx \frac{K_{a}Ks}{K_{a}Ks+1}$$

It is clear that the acceleration controller has a high gain Ka, thus the value of KaKs is large enough to keep certain gain of the closed loop at low frequency which is smaller than that of the ideal closed loop. The analysis will be verified by the subsequent experiments. The acceleration disturbance attenuation is as follows:

(10) $$\frac{\ddot{\theta }\left(s\right)}{{\ddot{\theta }}_{d}\left(s\right)}=\frac{1}{1+G_{a}\left(s\right)C_{a}\left(s\right)}$$

The velocity open loop response of the FSM with AFC at low frequency is depicted in Equation (11):

(11) $$G_{v\_a\_closed}=\frac{1}{s}G_{a\_closed}=\frac{K_{a}K}{\left(T_{1}s+1\right)\left(\frac{s^{2}}{{\varpi_{n}}^{2}}+\frac{2\xi }{\varpi_{n}}s+1\right)+K_{a}Ks}\approx \frac{K_{a}K}{K_{a}Ks+1}$$

Therefore, the traditional PI controller can meet the velocity closed-loop control. The disturbance attenuation of the two loop (velocity and acceleration loop) control system can be depicted as follows:

(12) $$\frac{\dot{\theta }\left(s\right)}{{\dot{\theta }}_{d}\left(s\right)}=\frac{1}{1+G_{a}\left(s\right)C_{a}\left(s\right)}\cdot \frac{1}{1+C_{v}\left(s\right)\frac{1}{s}\frac{G_{a}\left(s\right)C_{a}\left(s\right)}{1+G_{a}\left(s\right)C_{a}\left(s\right)}}$$

From Equation (12), we can see that the supersession bandwidth is subject to the acceleration closed-loop bandwidth. Comparing Equations (4) and (12), the disturbance attenuation with MEMS accelerometers and gyroscopes is better than that with FOG as long as the MEMS accelerometer bandwidth is higher than that of FOG.

**Figure 4:**
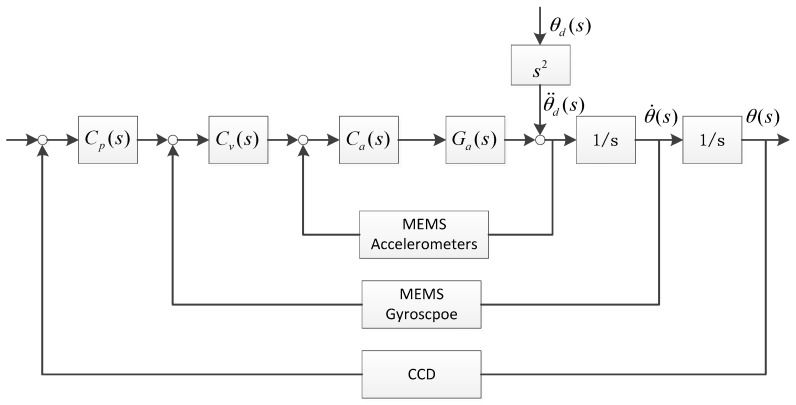
Stabilization control structure of multiple closed loops.

The multi-loop control structure of the FSM with the MEMS accelerometers and gyroscope is shown in Figure 4. For the FSM control system, the objective is to improve disturbance suppression performance.

Where *G_a_(s)* is the acceleration open loop transfer function, C*_a_(s)* is the acceleration controller, C*_v_(s)* is the velocity controller, C*_p_(s)* is the position controller, $\dot{\theta }(s)$ is the angular velocity output, $\theta_{d}(s)$ is the disturbance angle and ${\ddot{\theta }}_{d}(s)$ is the disturbance acceleration.

The disturbance suppression of the FSM with three closed loops can be expressed as follows:

(13) $$\frac{\frac{1}{s}\frac{G_{a}C_{a}}{1+G_{a}C_{a}}C_{v}}{1+\frac{1}{s}\frac{G_{a}C_{a}}{1+G_{a}C_{a}}C_{v}}\approx 1,\frac{G_{a}C_{a}}{1+G_{a}C_{a}}\approx 1$$

(14) $$\begin{array}{cc} E_{\theta } & =\frac{\theta (s)}{\theta_{d}(s)}=\frac{1}{1+G_{a}C_{a}+\frac{1}{s}G_{a}C_{a}C_{v}+\frac{1}{s^{2}}G_{a}C_{a}C_{v}C_{p}}\\ & =\frac{1}{1+G_{a}C_{a}}\cdot \frac{1}{1+\frac{1}{s}\frac{G_{a}C_{a}}{1+G_{a}C_{a}}C_{v}}\cdot \frac{1}{1+\frac{1}{s}\frac{\frac{1}{s}\frac{G_{a}C_{a}}{1+G_{a}C_{a}}C_{v}}{1+\frac{1}{s}\frac{G_{a}C_{a}}{1+G_{a}C_{a}}C_{v}}C_{p}}\\ & \approx \frac{1}{1+G_{a}C_{a}}\cdot \frac{1}{1+\frac{1}{s}C_{v}}\cdot \frac{1}{1+\frac{1}{s}C_{p}} \end{array}$$

Therefore, the disturbance suppression of the system is equal to the product of the individual suppression of three closed loops. 

For the velocity open loop transfer function without AFC, the function sensitivity is equal to 1. With AFC, the function sensitivity becomes:

(15) $$S_{G_{a}}^{{\dot{\theta }}_{a}}=\frac{\left(\frac{(G_{a}+\Delta G_{a})C_{a}C_{v}}{1+(G_{a}+\Delta G_{a})C_{a}}\cdot \frac{1}{s}-\frac{G_{a}C_{a}C_{v}}{1+G_{a}C_{a}}\cdot \frac{1}{s}\right)/\left(\frac{G_{a}C_{a}C_{v}}{1+G_{a}C_{a}}\cdot \frac{1}{s}\right)}{\Delta G_{a}/G_{a}}\approx \frac{G_{a}C_{a}}{{\left(1+G_{a}C_{a}\right)}^{2}}\approx \frac{1}{1+G_{a}C_{a}}$$

Providing that the gain of the acceleration controller is large enough, $S_{G_{a}}^{{\dot{\theta }}_{a}}$ is far less than 1. When the relevant structures and parameters of the system change greatly, the robustness of the velocity closed-loop system with AFC will not be affected. The gain of the acceleration controller actually exceeds 100, and so the system with the MEMS accelerometers and gyroscope feedback is robust.

## 3. Analyzing of the MEMS Accelerometers and Gyroscopes

**Figure 5:**
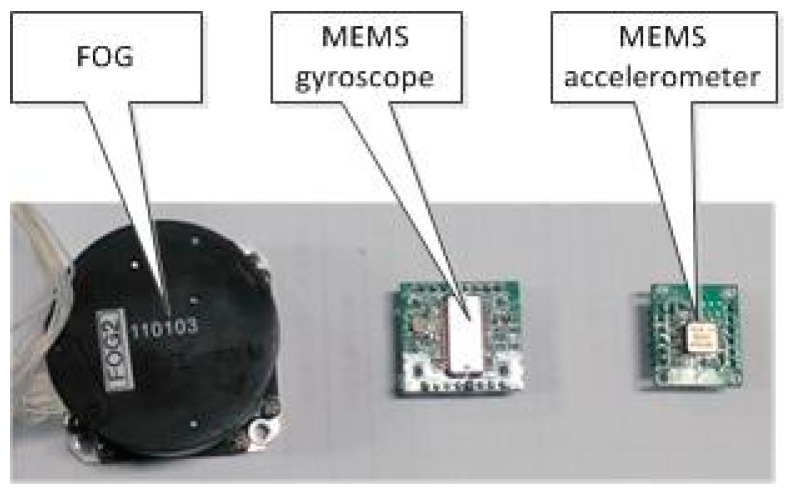
Picture of inertial sensors.

The MEMS accelerometer and gyroscope have smaller size, lower weight, lower price and lower power consumption than the FOG. Figure 5 and Table 1 depict the difference of the three types of sensors.

**Figure 6:**
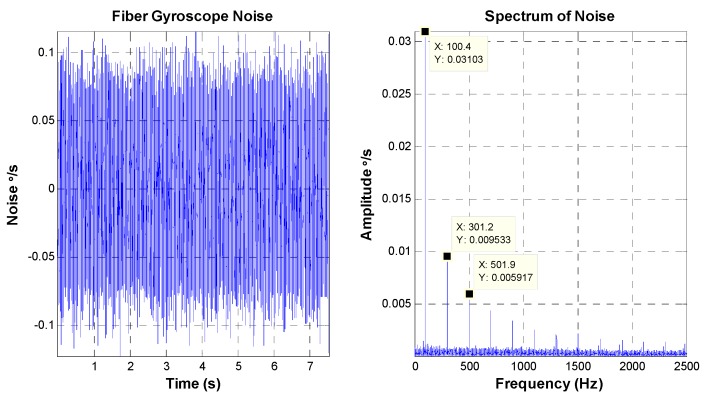
FOG noise characteristics.

The FOG-based Inertial Measurement Unit mounted on the mirror base is usually used as the feedback component of the velocity closed-loop in the high precision FSM control system. The bandwidth and noise of the FOG have significant effects on the performance of the FSM control system. The bandwidth of the FOG for the latter experiment is about 500 Hz. The characteristics of the FOG noise are depicted in Figure 6.

**Figure 7:**
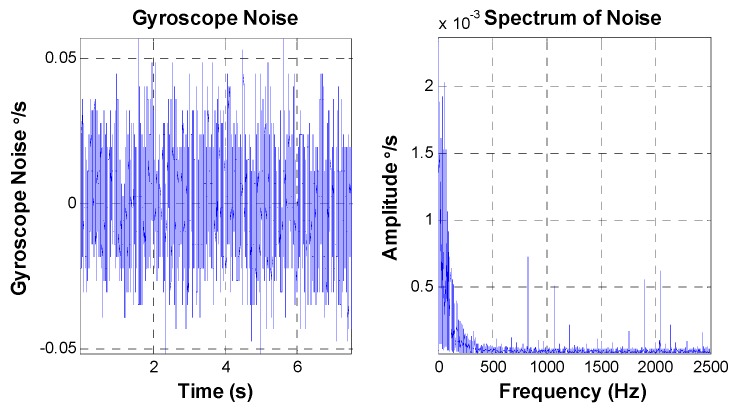
MEMS gyroscope noise characteristics.

The peak value of the FOG noise is equal to 0.25 °/s, and the RMS value is about 0.037 °/s. Aside from some “spikes”, the amplitude value of amplitude-frequency curve is smooth. The bandwidth of the MEMS gyroscope for the latter experiment is less than 100 Hz, which is lower than that of the FOG. Figure 7 shows the characteristics of the MEMS gyroscope noise.

The peak value of the MEMS gyroscope noise is equal to 0.12 °/s, and the RMS value is about 0.015 °/s, which is smaller than that of the FOG. The MEMS gyroscope noise maily foucus on the frequencies ranging from 0 to 200 Hz.

**Figure 8:**
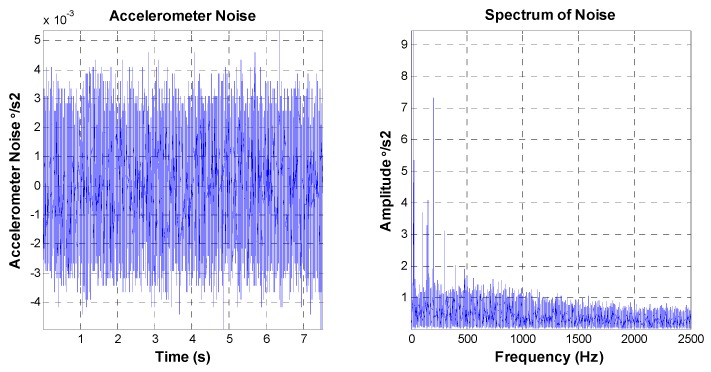
MEMS accelerometer noise characteristics.

The practical bandwidth of the MEMS accelerometer is about 1000 Hz, which is higher than that of the FOG. The characteristics of the noise are shown in Figure 8.

The peak value of the MEMS accelerometer transformed noise is equal to 0.01 °/s^2^, and the RMS value is about 0.00135 °/s^2^. The amplitude value of amplitude-frequency curve is mainly smooth aside from some “spikes”.

From the aforementioned analysis, the combination of MEMS gyroscopes and accelerometers can be used to replace the FOG for accurately measuring the FSM’s high bandwidth disturbance, and then the stabilized FSM with MEMS accelerometers and gyroscopes feedback can be accomplished to replace the strap-down FSM for the line-of-sight (LOS) stabilization. Furthermore, the price of the combination of two MEMS accelerometers and one MEMS gyroscope is 5000 Yuan, which is one-tenth of the price of one FOG.

## 4. Experimental Verification

**Figure 9:**
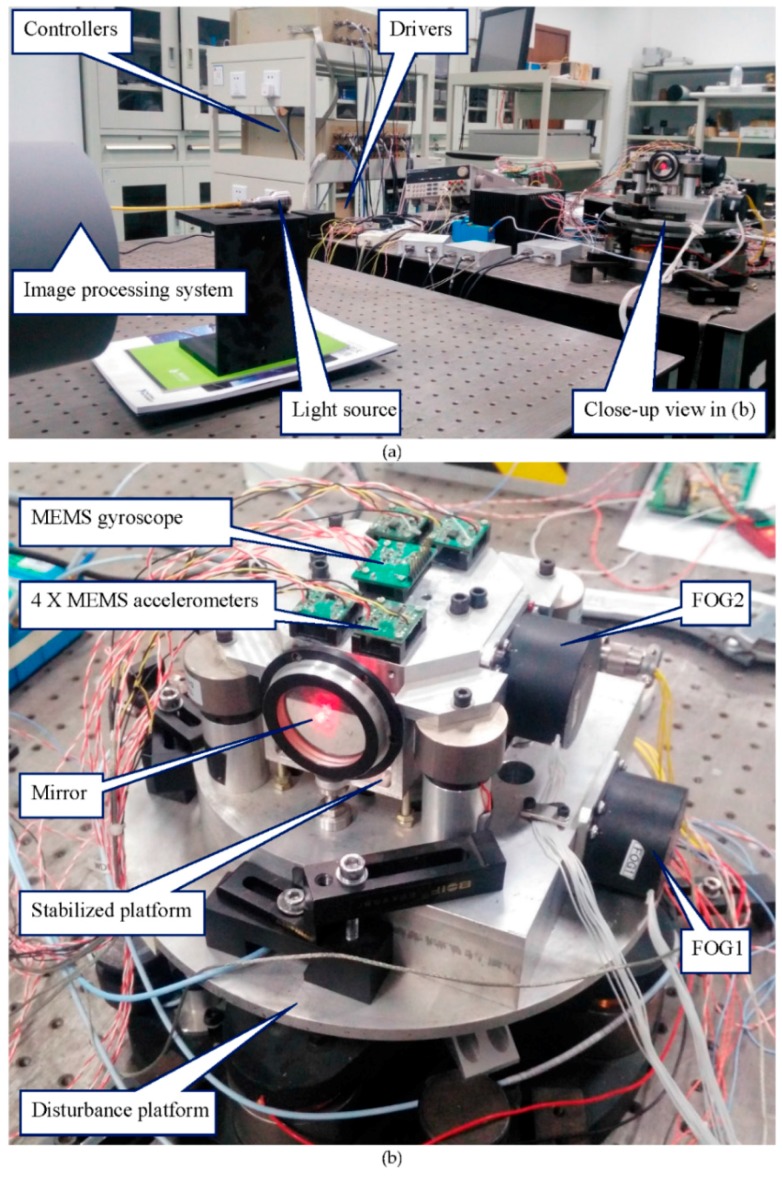
Experimental apparatus: (**a**) global diagram; (**b**) local diagram.

The FSM control system is a two-axis system. This experiment focuses on one axis due to the symmetry of the two axes. The experimental setup, which includes a disturbance platform, a stabilized platform, a laser light, and an image processing system (CCD), is shown in Figure 9. The two platforms are driven by voice coil motors. The disturbance platform simulates the disturbance of the carrier, on which the fiber-optic gyroscope1 (FOG1) and the eddy current displacement sensor are used for disturbance measurement, FOG1 is used to measure disturbance angular velocity of the platform, and the eddy is used to measure the disturbance angle of the platform. The stabilized platform is mounted on the disturbance platform. The MEMS linear accelerometers mounted on the stabilized platform are used to measure the angular acceleration of the platform. The fiber-optic gyroscope 2 (FOG2) is used to measure the angular velocity of the stabilized platform. The light source is used to simulate the target, and the mirror reflects the laser light into the image processing system which detects the LOS of the stabilized platform and provides LOS error. The schematic diagram and physical diagram are shown in Figure 9.

The MEMS accelerometer bandwidth exceeds 1000 Hz, and the angular acceleration can be obtained from two line accelerometers. The bandwidth of the FOG exceeds 500 Hz, and the bandwidth of the MEMS gyroscope is less than 100 Hz.

**Figure 10:**
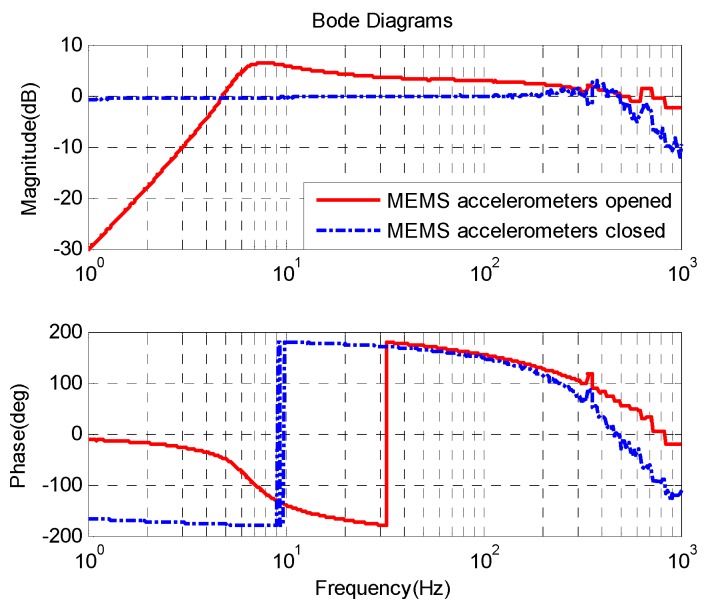
FSM acceleration response.

From Equation (5), the open loop acceleration characteristic of the FSM at low frequency is similar to a second-order integrator. Figure 10 shows the open loop and the closed-loop acceleration response. The acceleration closed-loop bandwidth exceeds 700 Hz and the closed-loop acceleration response includes a little differential effect at low frequency which is depicted in Equation (9). The static acceleration noise of AFC is about 0.0028 °/s^2^, which is about twice than the noise of MEMS accelerometers.

**Figure 11:**
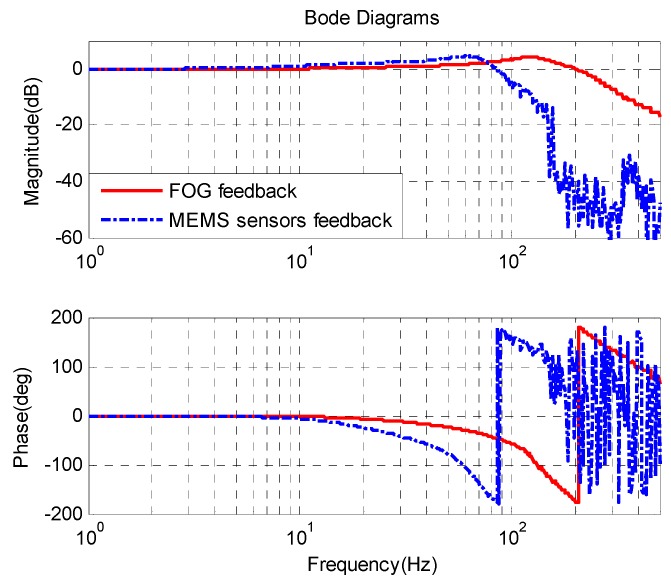
FSM velocity response.

The closed-loop bandwidth of the single FOG velocity loop is about 220 Hz in Figure 11, which is higher than that of the MEMS gyroscope velocity loop with MEMS accelerometer feedback. Therefore, the lower bandwidth will produce some adverse effect on the outer position loop. The static noise of the single FOG velocity loop is about 0.05 °/s, which is larger than that of the MEMS velocity loop with AFC. This is due to the different bandwidth of these sensors.

**Figure 12:**
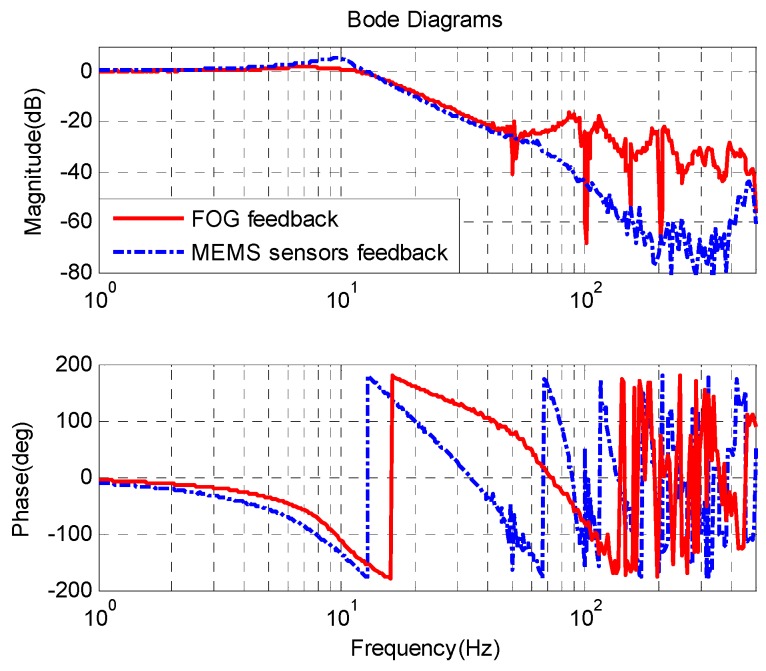
CCD closed-loop response.

Figure 12 shows the CCD closed-loop response of the velocity closed-loop FSM with FOG feedback and with MEMS sensors feedback. The sample rate of the CCD is 100 Hz, which limits the bandwidth of the CCD closed-loop. The closed-loop bandwidths of two kinds of systems all reach about 16 Hz. The static position error of the system with FOG feedback is approximately 0.113 pixel, while the static position error with MEMS sensors feedback is 0.115 pixel, which is a little larger than that with FOG feedback. The primary causes are electronic and sensor noises. 

**Figure 13:**
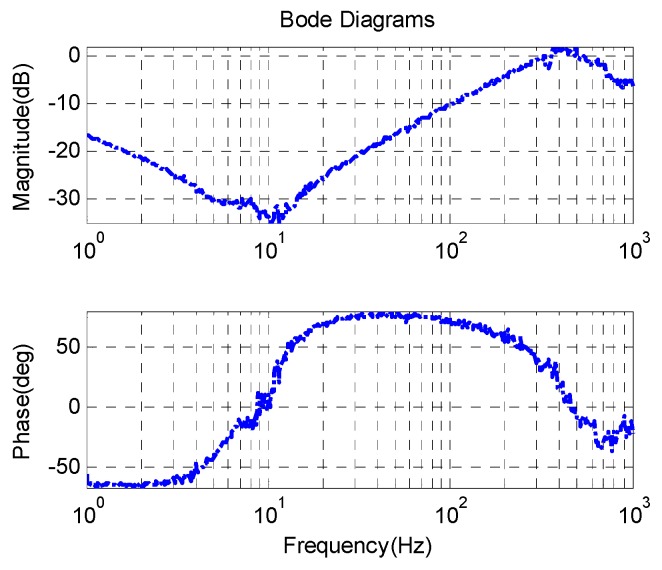
Disturbance suppression characteristics of the acceleration closed-loop.

It is observed from Figure 13 that the disturbance suppression of the MEMS accelerometer closed-loop at low frequency is weaker than at medium frequency because of the non-ideal acceleration controller *C^’^_a_* in Equation (5). Benefiting from the high closed-loop bandwidth, the MEMS accelerometer loop keeps the disturbance suppression ability in the range of 200 Hz.

**Figure 14:**
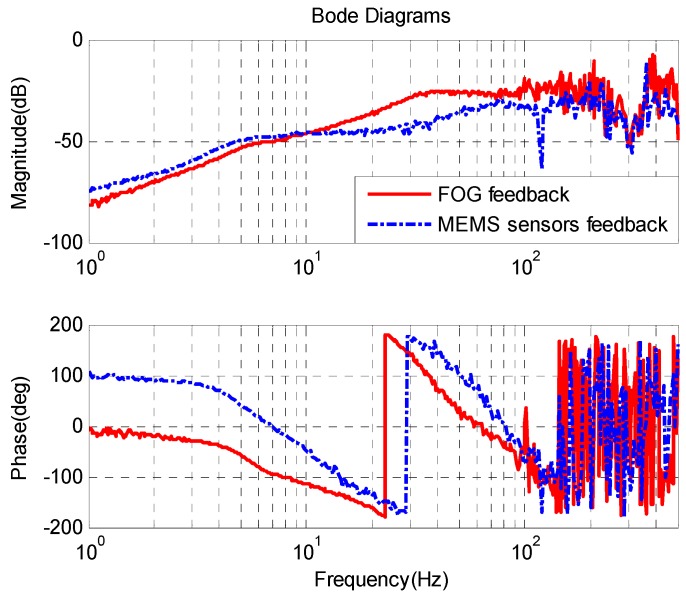
Disturbance suppression characteristics.

The disturbance suppression characteristics of the FSM control system with the CCD and gyroscope feedback are shown in Figure 14. The disturbance suppression with MEMS inertial sensors matches with that with FOG.

It is observed from Figure 14 and Table 2 that, with MEMS accelerometers feedback control, the disturbance suppression of the FSM multi-loop control system is improved. The experimental result accords with the aforementioned analyzing about Equations (4), (12) and (14).

The MEMS acceleration feedback loop has more than 700 Hz bandwidth (shown in Figure 10). However, the disturbance attenuation of the AFC is limited by the non-ideal acceleration feedback controller below 10 Hz. Therefore, the disturbance attenuation of the MEMS gyroscope feedback system with AFC is little weaker than that of the FOG feedback system below 10 Hz and is better between 10 to 100 Hz.

## 5. Conclusions

MEMS gyroscopes and accelerometers were introduced to replace the FOGs in the FSM control system. The modeling of the FSM acceleration via linear accelerometers was discussed from the viewpoint of its practical implementation. The simplification for implementing the AFC into the FSM control system was presented mainly in terms of the closed-loop stability and error attenuation. The algebraic expression shows that the AFC can effectively enhance the robustness of the closed-loop control system. Three types of inertial sensors was discussed in terms of size, weight, price, power consumption and performance. The experimental results showed that the MEMS accelerometers feedback can effectively enhance the stabilization performance of the closed-loop control system, and the combination of MEMS accelerometers and gyroscopes can act the role of the FOGs with lower cost in the FSM control system.

Future work will concentrate on reducing the cost of the FSM control system under conditions of good closed-loop performance. The use of only accelerometers may be an effective method, which will be our next work. The accelerometer signal may be processed by two-dimensional digital filters [15].

## Figures and Tables

**Table 1:** Comparison of the three types of sensors.

	FOG (FOG50)	MEMS Gyroscope (SCR1100-D02)	MEMS Accelerometer (Model 1221)
Size	Ø55 mm × 45 mm	25 mm × 20 mm × 8 mm	20 mm × 16 mm × 8 mm
Weight	250 g	25 g	20 g
Price	50,000 Yuan	1200 Yuan	1900 Yuan
Power consumption	6 W	0.15 W	0.1 W

**Table 2:** Comparison between disturbance suppressions of two kinds of FSM control systems.

Frequency	with FOG	with MEMS Inertial Sensors
1 Hz	−81.39 dB	−74.38 dB
10 Hz	−46.39 dB	−45.56 dB
20 Hz	−35.77 dB	−45.42 dB
40 Hz	−25.57 dB	−38.8 dB
100 Hz	−21.97 dB	−31.52 dB
